# Retraction Note: Optimization of dyes and toxic metals removal from environmental water samples by clinoptilolite zeolite using response surface methodology approach

**DOI:** 10.1038/s41598-022-22500-w

**Published:** 2022-10-19

**Authors:** Xinpo Sun, Reathab Abbass, Milad Ghoroqi, Indrajit Patra, Ngakan Ketut Acwin Dwijendra, Khusniddin Fakhriddinovich Uktamov, Hadeer Jasem

**Affiliations:** 1 College of Civil Engineering, Sichuan University of Science and Engineering, Zigong, 643000 China; 2 Medical Technical College, Al-Farahidi University, Baghdad, Iraq; 3 Department of Environmental Engineering, School of Environment, College of Engineering, University of Tehran, Tehran, Iran; 4 National Institute of Technology (NIT) Durgapur, Durgapur, West Bengal India; 5 Faculty of Engineering, Udayana University, Bali, 80361 Indonesia; 6 Economic Security Department, Tashkent State University of Economics, Tashkent, Uzbekistan; 7 Medical Instrumentation Techniques Engineering Department, Al-Mustaqbal University College, Babylon, Iraq

Retraction of: *Scientific Reports* https://doi.org/10.1038/s41598-022-17636-8, published online 02 August 2022

Editors have retracted this Article.

After publication, concerns were raised about authorship and description of author contributions. The Editors requested the authors to provide raw original data and explanations regarding the contributions, but found the response provided by the Authors insufficient. The Authors were also not able to provide the data in the format that would allow for the confirmation of its veracity (i.e. including sufficiently detailed meta-data). The Editors therefore no longer have confidence in the reliability of the data presented in this Article.

None of the Authors responded to the correspondence from the Editors about the retraction.

